# Supplementary material for: Identifying Quantum Structure in AI Language: Evidence for Evolutionary Convergence of Human and Artificial Cognition
Source: Entropy (Basel). 2026 Jun 1;28(6):622. doi: 10.3390/e28060622 (PMC13298953; doi:10.3390/e28060622)
Supplement: Supplementary file 1 [file entropy-28-00622-s001.zip › entropy-4269491-supplementary.pdf]

Supplementary Materials

Supplementary Materials for the article "Identifying Quantum Structure in AI Language: Evidence for Evolutionary Convergence of Human and Artificial Cognition" D. Aerts, J. Aerts Arguëlles, L. Beltran, S. Geriente, R. Leporini, M. Sassoli de Bianchi, and S. Sozzo.

In these Supplementary Materials, we present the additional stories analyzed in the study: a Winnie-the-Pooh story generated by ChatGPT, and a science-fiction story inspired by H. G. Wells's "The Magic Shop," generated by Gemini. We also provide the corresponding graphs of word frequencies and associated energy levels.

Details of the interpretation are given in the main article, where the analysis of the Winnie-the-Pooh story generated by Gemini is discussed. Excel files containing the full calculation details are available from the corresponding author upon request.

S1. A Winnie the Pooh story titled "In Which Tigger Wonders Where He Belongs and Everyone Tries to Help," written by ChatGPT.

In Which Tigger Wonders Where He Belongs and Everyone Tries to Help

One fine morning in the Hundred Acre Wood, the sun was smiling down through the trees as if it had just remembered a very funny joke. Birds were humming, bees were buzzing, and the leaves were rustling in a particularly rustly way. It was the sort of morning that made one think of jam, or adventures, or perhaps a little of both.

Tigger, who was bouncing, as Tiggers do, had stopped bouncing just for a moment. He stood quite still at the edge of a puddle, looking into it, and made a small noise like "Hmmmmph."

Now, a "Hmmmmph" from Tigger is not quite the same as a "Hmmmmph" from Eeyore. Eeyore's "Hmmmmph" means "I knew this would happen." Tigger's means something more like "Something is happening, and I'm not entirely sure what it is, but it might be very interesting indeed." Which is to say, it was a curious "Hmmmmph."

He looked into the puddle again and saw himself looking back. He made a face at himself. The reflection made the same face.

"Well, that proves *something,*" said Tigger. Then he sat down on a small log, which wobbled under him as if it wasn't used to holding someone quite so bouncy.

"I *say,*" said Tigger aloud to no one in particular, "what sort of animal *is* a Tigger, really?"

He had been wondering this ever since Roo asked him the day before if there were other Tiggers. And he had said "Of course!" very loudly, but when he got home he wasn't so sure.

"What if I'm the only one?" he had thought, and it made his tail feel a little less springy than usual.

So that morning, Tigger decided to find out. And when a Tigger decides something, it generally happens, sooner or later, or sometimes sideways.

First Stop: Pooh

Tigger's first visit was to Winnie-the-Pooh, who was humming a hum and tapping his head with a paw as if he were trying to get a thought to drop out.

“Hullo, Tigger,” said Pooh when he saw him. “Is it time for breakfast again?”

“Not just yet, old buddy,” said Tigger. “I came to ask a Very Important Question.”

Pooh stopped tapping and looked thoughtful, which for Pooh took some effort.

“Do you know,” asked Tigger, “what kind of animal a Tigger is?”

Pooh blinked.

“You’re a Tigger,” said Pooh.

“Yes, *but what sort of* Tigger?” said Tigger. “I mean, where do Tiggers come from?”

Pooh scratched behind his ear.

“Do they come from jars?” he asked helpfully. “I know honey comes from jars, although before that it comes from bees, and before that it comes from flowers.”

“I don’t think I came from a flower,” said Tigger, slightly worried.

“Maybe ask Owl,” said Pooh. “He knows about things like that. He has a book and everything.”

So Tigger thanked Pooh with a small bounce, just enough to make Pooh sit down suddenly, and he bounded off toward Owl’s house.

Owl Has Ideas

Owl was perched outside his door, rearranging his thoughts.

“Hullo Tigger,” he said. “What a propulsive morning!”

“I came to ask a Very Scholarly Question,” said Tigger.

Owl swelled a little with pride.

“Oh, splendid,” said Owl. “Fire away, dear fellow.”

“What sort of animal is a Tigger?”

Owl adjusted his spectacles, although he wasn’t wearing any.

“Well,” he began, “the name ‘Tigger’ bears a striking resemblance to *tiger*, which is a large, stripy feline found in faraway lands. But of course, you have a rather more exuberant disposition than the average tiger.”

“Are they bouncy?” asked Tigger, interested.

“No,” said Owl. “Quite the opposite. Rather stealthy. Predators, you know.”

“Oh,” said Tigger, deflating slightly.

“But!” said Owl. “There’s nothing to say that you couldn’t be a most unique and singular specimen of a New Type of Animal, which we shall call a *Tiggerius Springibundus.*”

“Springy-bun-what?” said Tigger.

“Latin,” said Owl. “It means ‘bouncy and splendid.’”

Tigger perked up.

“Then I *am* something special!”

“Quite,” said Owl. “Now, if you’d care for a brief lecture on the genealogy of exotic quadrupeds—”

But Tigger had already bounced away again.

A Visit to Kanga and Roo

Next, Tigger visited Kanga and Roo. Roo ran out to meet him, bouncing almost as high as Tigger, which made Tigger very proud.

“Tigger!” cried Roo. “Did you find other Tiggers?”

“Not yet,” said Tigger. “But I found out I’m special. I might be a *Tiggerius Springy-something*.”

“Can I be one too?” asked Roo.

Kanga, who had been watching fondly from the doorway, said gently, “Everyone is special, Roo, but everyone is also *themselves.* That’s the important thing.”

“I think I might be the only one,” said Tigger, after a pause. “The only Tigger.”

“Is that a sad thing?” asked Kanga.

“I don’t know,” said Tigger honestly.

“Well,” said Kanga, “sometimes being the only one can be lonely. But it can also mean that you’re loved in a way no one else could be.”

Tigger blinked.

“Do you think the others like me because I’m me?”

“I do,” said Kanga, and Roo nodded so hard he fell over.

Tigger smiled a very small, very real smile.

“Thanks,” he said, and bounded away more quietly than usual.

Tigger and the Thousand Acre Forest

Tigger bounced and bounced, but each bounce felt a little slower than the last, like thoughts weighing down his spring. Past Owl’s house and past the stream, where pebbles giggle under the water, he wandered into the deeper forest—not the Hundred Acre Wood, but something beyond, older perhaps, and less visited.

It was what some might call the Thousand Acre Forest.

Nobody called it that, not officially, because nobody quite knew how large it was. But it was the sort of place that, when you entered it thinking, made your thoughts longer, slower, and more curious. Trees stood tall and thoughtful. Breezes whispered instead of whooshing. Everything seemed to listen more than it spoke.

And so Tigger, lost in a great pondering of what it meant to be a Tigger—and to be the only one—found himself among very tall trees, where the sun filtered in through high branches in patterns that looked a little like poetry.

“Oh bother,” said Tigger, which is something he sometimes said when he forgot he wasn’t Pooh.

He sat at the foot of an old oak tree and looked up.

“Excuse me,” he said politely to the tree. “Do you know what sort of animal a Tigger is?”

The oak said nothing, but its branches swayed gently, like a nod in slow motion.

“Are you saying you know? Or you don’t know? Or you’re just being a tree?” asked Tigger.

A leaf spun down and landed on his nose. Tigger sneezed. Then he giggled. Then he sighed.

"I suppose you're going to say something like, 'A tree is a tree because it grows, and it's itself no matter who else is around.' But that's easy for you. There are loads of oaks. You've got your acorns and your roots and all. But I'm just one. Just me."

Another leaf floated down. Tigger caught it gently in a paw. He looked at it.

"This leaf isn't you," he said. "But it's still part of you."

And then he frowned in that way Tiggers frown when they're not used to their own thoughts making sense.

"Am I a leaf?" he wondered aloud. "Or a tree? Or maybe both?"

The forest didn't answer, but a breeze rustled around him as if saying, "Keep going."

So Tigger did.

He bounced a little further, slowly, and came to a patch of wildflowers—bluebells, buttercups, and tall thistles, which he avoided carefully because he was still feeling polite after Eeyore. The flowers stood in soft, colourful clusters, and bees moved among them like they had very important appointments to keep.

Tigger lay down in the middle of them, not on the flowers, but near enough to smell them.

"Hullo bees," he said. "Do you know what I am?"

The bees buzzed past his ears and didn't stop, but one paused on a buttercup and waggled its back end very precisely.

"I don't speak Bee," said Tigger, "but that looked like dancing. So maybe you're saying I should dance my way through life? Bounce and bloom and buzz?"

The bee ignored him and flew away.

"Typical," muttered Tigger, and lay back, paws behind his head, tail curled like a question mark.

For a long time, he didn't say anything. He just watched the sky move between the branches, watched the ants trail across moss, watched how everything was busy being exactly what it was.

The grass grew by growing. The birds flew by flying. The clouds floated by simply being clouds.

Nobody seemed confused about who they were. Not even the mud.

"Maybe," he thought, "being the only one isn't the same as being the wrong one."

That thought settled in his tummy like a warm biscuit.

And then, after a while, he started talking out loud—not to anyone in particular, but to the forest itself, which was very good at listening.

"I don't roar like a tiger. I don't hoot like an owl. I don't hum like Pooh. I don't hop like Roo, or sit like Eeyore, or make tea like Rabbit. I bounce. And I talk too much. And I make a mess sometimes. But I also hug very hard. And I laugh with my whole body. And sometimes I wake up in the middle of the night and think, 'Is this it? Am I finished being made?'"

He paused.

"Maybe being a Tigger means never quite being finished. Always wondering. Always bouncing into something new."

A pair of birds chirped in a low branch above him.

"Does that sound silly?" he asked them.

They chirped again.

"I suppose everything sounds a bit silly when you say it out loud in a forest."

The sky turned golden as afternoon began to think about becoming evening. Tigger stood up, stretched, and looked around.

"I've been thinking so hard I forgot to think about going home."

But just then, the forest did something very kind. It shifted a little—just a little—like a path folding open. A breeze moved the leaves in such a way that the way back became clear.

"Thanks," said Tigger to everything—the trees, the bees, the flowers, the birds, even the silent sky. "I think I needed to get a little lost to remember what it means to be found."

He bounced—just once, experimentally—and felt the spring return to his tail. A little slower than usual. A little steadier.

Then he said it aloud:

"I'm Tigger. The only one. And that might just be exactly the right number."

And the forest rustled as if it agreed.

Piglet Tries to Help

Piglet was out collecting small things when Tigger landed beside him in a poof of leaves.

"Eep!" said Piglet.

"Don't be frightened, Piglet! It's only me!" said Tigger.

"Oh," said Piglet, patting his chest. "That's all right then."

Tigger explained his problem.

"Well," said Piglet, "I suppose you're the sort of animal who makes things more exciting."

"Exciting?"

"Yes. You bounce in, and things happen."

"Like what?"

Piglet thought.

"Like someone ends up in a tree, or covered in flour, or sometimes a little dizzy."

"That doesn't sound very good," said Tigger.

"But it *is!*" said Piglet. "Because you make things lively. And when you're not there, it's quieter, but also a little more—" he paused, "*less*."

Tigger had never thought of it like that.

"Thanks, Piglet," he said, and gave him a careful pat on the head. Piglet beamed.

Eeyore's Opinion

Last of all, Tigger visited Eeyore, who was contemplating a thistle.

“Hullo Eeyore,” said Tigger.

“Don’t mind me,” said Eeyore. “Just sitting here. Thistle’s doing fine. Nobody asked, but it’s managing.”

“I wanted to ask you something,” said Tigger.

“Strange day,” said Eeyore. “People asking Eeyores questions.”

“I’ve been wondering,” said Tigger, “what kind of animal I am. Owl says I’m a *Tiggerius Springy-whatever*, and Roo thinks I’m the only one, and Kanga says that makes me special, and Piglet says I make things exciting.”

Eeyore sniffed.

“Sounds like you’ve got it all figured out.”

“But I still don’t know where I belong.”

Eeyore looked at him.

“You belong,” he said, “in the Hundred Acre Wood. Because you’re here. That’s usually how it works.”

Tigger blinked.

“Oh,” he said.

Then he sat beside Eeyore, just for a while, and neither of them said anything more.

A Gathering at Pooh Corner

That evening, as the sun was falling down behind the trees like honey slipping off a spoon, Christopher Robin came to visit.

He found them all gathered at Pooh’s house: Pooh with a sticky paw, Piglet with a very small leaf, Kanga and Roo sitting close together, Owl explaining something complicated to no one in particular, and Eeyore chewing thoughtfully. Tigger was lying on his back, looking at the sky.

“Hello, everyone,” said Christopher Robin.

“Hullo,” they said.

“Tigger’s been wondering who he is,” said Pooh.

“Still am,” said Tigger.

Christopher Robin sat down next to him.

“Well,” he said, “you’re Tigger. The one and only.”

“But what *does* that mean?” asked Tigger. “Am I a tiger? Am I just bouncy? Am I an accident?”

Christopher Robin looked thoughtful.

“You’re *you,*” he said. “The one who makes everyone laugh. Who bounces at just the right time, even if it’s the wrong time. You’re the friend who turned Roo upside down and made him giggle. Who made Piglet brave by accident. Who made Eeyore admit he liked company. Who made us all realize that sometimes, you don’t need to *know* who you are to be important.”

Tigger looked at him for a long time.

“That sounds like a lot.”

“It is,” said Christopher Robin. “And you do it just by being Tigger.”

Tigger thought. Then he smiled.

“Well then,” he said. “I suppose I *am* a very good sort of Tigger after all.”

And everyone agreed.

And the sun dipped the last bit below the trees, and they sat in the warm dusk, the best kind of silence there is: the kind that’s full of friends.

The End

(Or, as Tigger might say, “The End! The End! Let’s read it again!”)

S2. Charts Illustrating the Data Analysis of the Winnie-the-Pooh Story Generated by ChatGPT

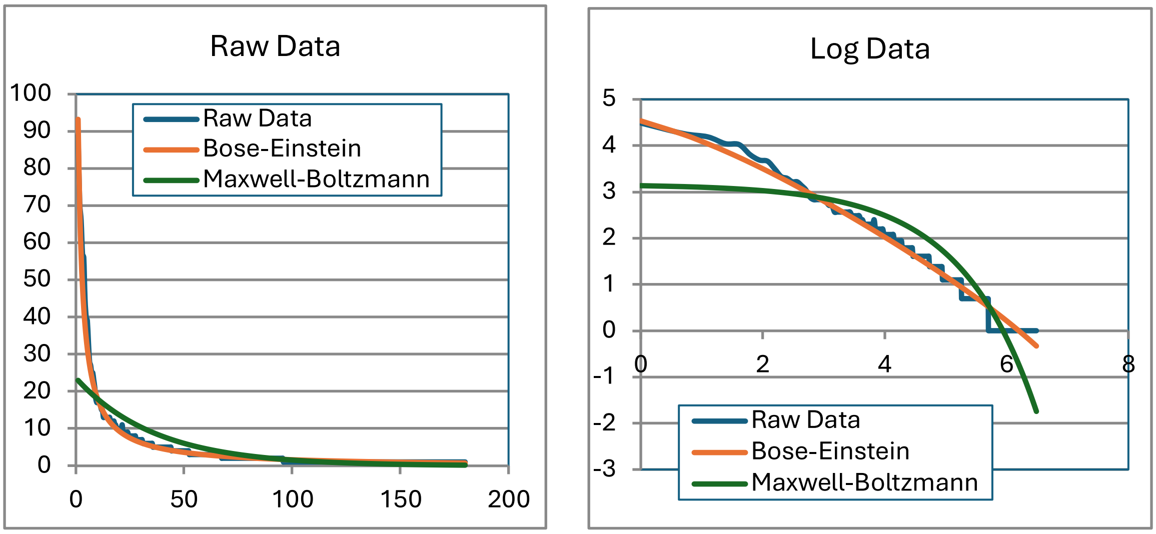

(a) **(b)**

Figure S1. (a) The 'numbers of appearances' $N(E_i)$ of words in the Winnie the Pooh story written by ChatGPT ranked from the lowest energy level, corresponding to the most frequently appearing word, to the highest energy level, corresponding to the least frequently appearing word. The blue line represents the data, i.e. the numbers of appearances deduced from the story, the red line represents the numbers of appearances predicted by the BE distribution model, and same for the green line but for the MB distribution model. **(b)** The same data but using a log-log plot (both axes on a logarithmic scale). The red and blue line coincide almost completely in both graphs, whereas the green line does not coincide at all. This shows that the BE distribution is a good model for the numbers of appearances, while the MB distribution is not. We have $d = 0.8$.

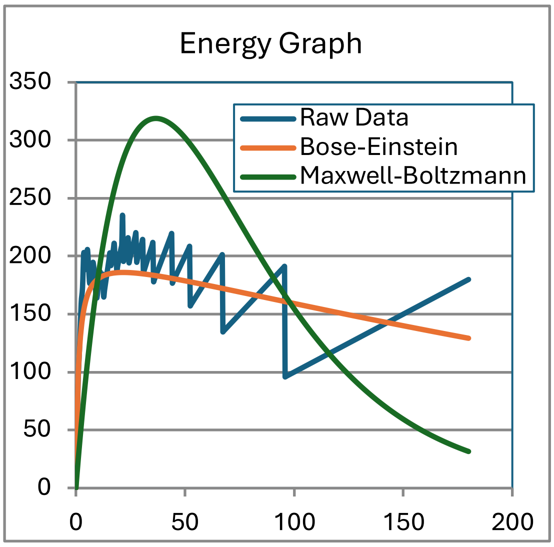

Figure S2: The energy $E(E_i)$ radiated per energy level of the Winnie the Pooh story written by ChatGPT as a function of the energy levels E_i. The blue line represents the data, the red line represents the values predicted by the BE model, and same for the green line but for the MB model. We have $d = 0.8$.

S3. A science fiction story titled “The Atelier of Acausal Curiosities,” written by Gemini

Dr. Alistair Finch considered himself a custodian of order. His days, spent navigating the labyrinthine datasets of a multinational tech firm, were a testament to the predictable march of logic, the elegant decay of uncertainty into digestible probabilities. Entropy, to Alistair, was a quantifiable nemesis, a force to be wrestled into submission by algorithms, its chaotic whispers silenced by the crisp, clean lines of a wellstructured spreadsheet. He understood the Second Law of Thermodynamics as an immutable decree, a universal truth as rigid as the very code he wrote. But the city, even his meticulously mapped corner of it, had a way of resisting absolute order.

One blustery Tuesday, the kind where the wind seemed to whisper non-sequiturs down the cobbled streets, Alistair found himself, and more importantly, his precocious nine-year-old niece, Elara, unexpectedly diverted. They’d been aiming for the celebrated patisserie on Elm Street, but a sudden downpour had forced them into the dubious shelter of a narrow alleyway. And there, where no shop had been yesterday, certainly no shop listed on any of Alistair’s geo-referenced apps, stood “The Atelier of Acausal Curiosities.” The storefront was anachronistic, an island of dark wood and frosted glass amidst modern steel and chrome. Its display window held not a single item of discernible purpose, but rather shimmering, iridescent voids that seemed to warp the light around them, and objects—perhaps mere dust motes—that subtly shifted their form when not directly observed, like peripheral thoughts that solidified only when ignored. “Oh, look, Uncle Alistair!” Elara’s voice, a bright chime against the city’s roar, pulled him from his bewildered contemplation. Her small finger, sticky from a half-eaten lollipop, pointed decisively at the shop’s entrance. “It looks like a magic shop!” Alistair snorted, a dry, data-driven sound. “Nonsense, Elara. There’s no such thing as magic. Just misdirection, sleight of hand, or, more likely, a very clever marketing gimmick.” Yet, a flicker of something he couldn’t quite categorize—a statistical anomaly in his otherwise predictable day—nagged at him. The absence of the shop from his digital cartography was, frankly, infuriating. Before he could articulate his skepticism further, the heavy wooden door creaked open, seemingly of its own accord. A figure emerged, neither welcoming nor forbidding, simply present. The shopkeeper was ageless, their features smooth and unlined, yet their eyes held the depth of ancient, settled starlight. They wore simple, dark attire that seemed to absorb the light rather than reflect it.

“Welcome,” the shopkeeper said, their voice a low, resonant hum, like a perfectly struck tuning fork. It seemed to bypass Alistair’s auditory nerves and settle directly into the pre-frontal cortex.

“You are precisely of the right sort. Please, step inside.”

Alistair hesitated, a surge of professional caution warring with a peculiar, almost academic, curiosity.

“Of the right sort for what, precisely?” he asked, trying to inject a note of informed skepticism into his voice.

The shopkeeper offered a faint, knowing smile. “For understanding, perhaps. For witnessing the true economy of information. For appreciating the elasticity of causality, unburdened by... conventional interpretations of disorder.”

Elara, already halfway through the doorway, tugged impatiently at his coat. “Come on, Uncle Alistair! It’s an Atelier ! They make things!”

Resigning himself, Alistair stepped across the threshold, the familiar drone of the city instantly muffling behind him. The air within the Atelier was cool, almost sterile, yet subtly charged,

like the moment before a storm. The interior was not cluttered with shelves or trinkets, but minimalist, almost ascetic. Three pedestals stood in the center, each bearing a single, enigmatic object. This wasn't a shop. It was, as its name suggested, a workshop, or perhaps, a gallery for concepts.

"My apologies for the... unconventional appearance," the shopkeeper intoned, gesturing to the three objects. "We deal not in illusions, but in revelations. In challenging the ingrained prejudices of what can be. You, Dr. Finch, are a man of data. You understand that information is fundamental. But perhaps you have yet to grasp its true malleability, its inherent capacity for rearrangement."

Alistair felt a prickle of annoyance. "Information is subject to the laws of thermodynamics, just like everything else. It degrades. It spreads. That's entropy."

The shopkeeper's smile widened imperceptibly. "Indeed. But what if the very form of

information could be manipulated? What if its 'decay' could be inverted, or its 'spread' precisely focused?"

They moved towards the first pedestal, drawing Alistair's attention to a small, intricate device resembling a polished, multi-faceted crystal. It pulsed with a faint, inner luminescence.

"This," the shopkeeper announced, "is the Entangled Effigy."

Alistair leaned closer to the Entangled Effigy, his data scientist's eye instinctively searching for seams, wires, any tell-tale sign of a hidden mechanism. The crystal was flawless, humming with a barely perceptible vibration that seemed to tickle his fingertips even before he touched it.

"Observe," the shopkeeper murmured, placing a small, sealed glass vial onto a recessed platform beside the effigy. Inside the vial, a colorless liquid shimmered, tiny thermal currents visibly swirling within it. Alistair's internal thermometer registered it as slightly warmer than room temperature – a system with its own minute, chaotic dance of molecular energy.

The shopkeeper then placed a miniature, intricately designed turbine, no larger than his thumb, on another small stand connected by a thin, almost invisible filament to the effigy's base. "This liquid," they explained, their voice a silken thread of sound, "possesses a certain degree of thermal energy, its molecules in a state of high entropy. Traditionally, extracting usable work from such a system without introducing an even greater disorder elsewhere is. . . inefficient, to say the least. The Second Law, as you know."

Alistair nodded, a professional reflex. "Irreversible. You'd need a colder reservoir, a temperature differential, and even then, you're always losing energy as waste heat."

"Precisely," the shopkeeper conceded, a hint of something like amusement in their eyes.

"Unless, of course, you could manipulate the information within the system. The very informational structure of its constituent particles."

With a slow, deliberate movement, the shopkeeper laid a hand on the Entangled Effigy. No buttons were pressed, no switches flicked. A soft, inner light within the crystal intensified, pulsing rhythmically. Alistair watched, utterly transfixed, as the liquid in the vial began to clear. The thermal currents that had been so evident moments before visibly diminished, then vanished entirely. The glass of the vial, which had been warm, felt noticeably cool to Alistair when he tentatively touched it. And then, impossibly, the tiny turbine beside it began to spin. Slowly at first, then with increasing speed, its delicate blades a blur, generating a faint, high-pitched whine. It continued to spin, drawing mechanical work from the cooling liquid, a silent,

irrefutable defiance of everything Alistair understood about energy conversion and the relentless march of entropy.

"What. . . what is this?" Alistair stammered, his mind racing, trying to categorize the anomaly. It couldn't be a simple Peltier effect, nor any known thermoelectric principle. The scale was wrong, the efficiency absurd.

"We are creating entangled states within the system," the shopkeeper explained, their voice devoid of triumph, merely stating a fact. "By establishing and then selectively collapsing these quantum correlations, we precisely extract ordered information from the inherent chaos of the thermal bath. This isn't a reversal of entropy, Dr. Finch. It's a localized, highly controlled diminution of von Neumann entropy, a meticulous rearrangement of informational probability space. The 'cost' is not borne by a thermal exhaust, but by the re-sculpting of the informational landscape itself, perhaps shifted to a realm beyond your current perception."

Elara clapped her hands, her eyes wide with delight. "It's like a tiny engine that runs on. . . nothing!"

Alistair barely registered her. His thoughts spun faster than the turbine. If what he was witnessing was genuine, if you could truly pull usable energy from ambient heat simply by manipulating quantum information, the implications were cataclysmic. Energy crises, resource depletion, the very foundations of industrial civilization – all rendered moot. But also, the terrifying thought: what else could be rearranged? What other fundamental laws could be bent, not through brute force, but through elegant, invisible informational architecture?

The shopkeeper withdrew their hand, and the crystal's light dimmed. The turbine slowed, then stopped. The liquid in the vial slowly began to regain its ambient temperature, the subtle currents reappearing. The demonstration was over, leaving behind only the cold, hard fact of what Alistair had witnessed. His perfectly ordered world, secured by the unshakeable certainty of the Second Law, now felt as fluid and unpredictable as the shimmering voids in the shop's front window.

"Fascinating," Alistair managed, the word tasting like ash in his mouth. His data, his algorithms, his entire professional life, seemed utterly trivial in the face of such a profound, almost blasphemous, manipulation of reality.

"That," the shopkeeper replied, a knowing glint in their ancient eyes, "was merely a prelude. We have other curiosities that delve deeper into the nature of information, causality, and the very architecture of thought itself."

Alistair, still reeling from the impossible spectacle of the Entangled Effigy, found himself drawn, almost against his will, to the next pedestal. His mind, trained for decades to find patterns and logical sequences, now churned with the effort of reconciling what he'd just witnessed with the bedrock of his scientific understanding. It was like trying to fit a fractal into a perfectly straight line.

"And this," the shopkeeper announced, gesturing to a polished, antique pocket watch resting on a velvet cushion, "is the Chronos-Entangler." The watch, devoid of any visible hands, shimmered with a faint, internal light, its surface occasionally rippling as if reflecting an unseen current.

Elara, who had been quietly examining the now inert turbine with a child's unburdened curiosity, peered at it intently. "It doesn't have any numbers, Uncle Alistair. How does it tell time?"

"It tells a time, young Elara," the shopkeeper corrected gently, "but not in the linear fashion you're accustomed to. It tunes into the conceptual fabric of causality, where thought-forms, in

their Bose-Einstein (BE) states, can be accessed non-linearly, transcending the material arrow of time."

Alistair scoffed, a nervous habit. "Conceptual fabric? Bose-Einstein condensates are physical states,exceedingly fragile, requiring near-absolute zero temperatures. You're speaking in metaphor."

"And you, Dr. Finch, are speaking of observation limited by conventional tools," the shopkeeper replied, their voice betraying no impatience. "Thought, in its nascent form, carries its own energy and entropy. These are not ephemeral neural firings alone, but structured informational entities. This device merely allows us to perceive their BE form, much like a radio receiver picks up a specific frequency."

With a fluid motion, the shopkeeper lifted the Chronos-Entangler and offered it to Alistair. "Hold it. Allow it to resonate with your own thought-stream. Focus on a decision, perhaps, or a moment of uncertainty from your past. Or even a fleeting intuition about the future you haven't yet formalized."

Hesitantly, Alistair took the watch. It was surprisingly light, cool to the touch, yet immediately he felt a subtle thrumming against his palm, almost like a faint pulse. He focused, as instructed, on a recent, complex decision he'd made at work – a contentious data migration strategy that had consumed weeks of his life. He'd chosen one path, confident in his statistical models. As he concentrated, the watch face, previously blank, began to shimmer intensely. Instead of numbers, intricate, shifting patterns of light and shadow flickered across its surface. Alistair found his mind suddenly flooded, not with images of the past, but with a vivid, visceral conceptual echo of an alternate decision. He saw, with chilling clarity, how a different, seemingly insignificant piece of data, overlooked in his original analysis, could have led to an entirely different, perhaps more efficient, migration strategy. It wasn't a memory; it was a parallel possibility, a road not taken, presented as a fully formed conceptual reality, complete with the emotional resonance of that hypothetical choice. Then, just as suddenly, the patterns dissolved, replaced by a disorienting flash of something else entirely. It was a fleeting, abstract 'tune,' a sense of a future thought-form he hadn't yet conceived. A specific sequence of algorithms, a novel approach to anomaly detection that felt utterly alien, yet undeniably right. It felt like a whisper of a future breakthrough, a solution to a problem he hadn't even fully articulated in his conscious mind. It was a conceptual arrival, an idea that felt pre-ordained yet utterly new. He dropped the watch as if it had burned him, his breath catching in his throat. His entire body trembled. "That. . . that wasn't a memory," he whispered, his voice hoarse. "It was. . . a possibility. And. . . a premonition."

"Indeed," the shopkeeper affirmed, retrieving the watch with serene grace. "You experienced a 'conceptual entanglement.' Your mind, in its BE form, momentarily touched upon informational pathways outside your conventional temporal perception. We are not dealing with the past or future of material events, Dr. Finch, but the energy and entropy of pure thought, untethered from its linear progression. We are 'tuning' into the conceptual world, where the arrow of time holds less sway."

Elara, wide-eyed, pointed. "Uncle Alistair, your face is all squishy!"

Alistair barely heard her. His reality, already fractured by the Entangled Effigy, was now shattering entirely. If thoughts, decisions, even future ideas existed in some pliable, non-linear informational state, what did that mean for free will? For the unique, unrepeatable trajectory of one's own life? His carefullyconstructed world of linear causality and quantifiable outcomes was dissolving into a dizzying vortex of infinite, simultaneous possibilities. He felt a profound, chilling sense of being adrift in an ocean of information,

where even his most private thoughts were not truly his own, but echoes in a vast, interconnected conceptual space. Alistair's breath was shallow, his heart hammering against his ribs. The cold dread that had settled in his stomach with the Entangled Effigy had now blossomed into a full-blown existential terror after his encounter with the Chronos-Entangler. His understanding of time, of decision, of even the very linearity of his own existence, felt profoundly violated. He watched, almost in a trance, as the shopkeeper moved to the third, and final, pedestal. Upon it rested a simple, unadorned glass sphere, filled with what appeared to be ordinary water. It seemed utterly innocuous, a stark contrast to the shimmering complexities of the previous devices. Yet, after what he had just experienced, Alistair knew better than to trust appearances within the Atelier.

"And this," the shopkeeper began, their voice maintaining its unnervingly calm resonance, "is the Mnemic Condenser." They gestured towards the sphere. "You, Dr. Finch, are accustomed to your thoughts being ephemeral, confined to the neural pathways of your brain. Yet, every thought, every memory, is an informational construct. It carries its own unique energy and entropy, and crucially, its own distinct Bose-Einstein (BE) form."

Alistair forced himself to speak, his voice a strained rasp. "You're suggesting thoughts are. . . physical entities? That they can be externalized?"

"Not physical in the sense of mass or conventional energy," the shopkeeper corrected patiently, "but real as information. This device allows us to observe the pure informational architecture of consciousness, before it's constrained by neurological pathways. It acts as a direct thought-form accumulator and projector." With a precise movement, the shopkeeper gently placed their palm against the glass sphere. For a moment, nothing happened. Then, slowly, almost imperceptibly, intricate, shimmering patterns began to form within the water. They weren't ripples from movement, but complex, crystalline structures of light and shadow, constantly shifting, evolving, and dissolving. They seemed to dance with an impossible, internal logic.

"Focus, if you will, on a specific memory," the shopkeeper instructed Alistair. "A vivid one. Observe how the sphere responds."

Alistair, despite his profound unease, found himself compelled to comply. He closed his eyes for a moment, recalling a childhood memory: the taste of his grandmother's homemade apple pie, the warmth of her kitchen, the specific pattern of light falling through the window. It was a sensory-rich, deeply nostalgic thought. When he opened his eyes and looked at the Mnemic Condenser, the patterns within the water had coalesced into a more defined, albeit still abstract, form. It wasn't a literal image of the pie or the kitchen, but a complex, oscillating geometry of light that, uncannily, felt like the memory. It carried the same warmth, the same specific essence of that moment. He felt a profound, almost invasive, connection to the patterns, as if his very internal experience was being made visible.

"You are witnessing the BE form of your thought," the shopkeeper explained. "Its unique informational signature, now externalized. This 'condensation' of your mental energy allows us to analyze its entropic state, its informational density. And, with sufficient focus..." The shopkeeper paused, their eyes locking with Alistair's. "It can also be used to project a concept. To resonate a desired thought-form within the mind of another, subtly influencing their own neural pathways by injecting this condensed informational packet." As they spoke, the patterns in the water intensified, pulsing with a new, insistent rhythm.

Alistair felt a subtle pressure behind his eyes, a strange, creeping sensation of a thought not his own beginning to take root in his mind. It was a concept of profound resignation, an acceptance of the inevitable decay of all complex systems, a surrender to the overwhelming forces of

chaos. It was the antithesis of his life's work, yet it felt strangely compelling, almost comforting in its finality.

He recoiled sharply, stumbling back from the pedestal, shaking his head to clear the invading thought.

"No! That's. . . that's an invasion. A violation of consciousness."

The shopkeeper merely inclined their head. "Is it, Dr. Finch? Or merely a demonstration of the true, interconnected nature of information? If thoughts are merely highly organized informational structures, with their own energy and entropy, then perhaps their 'privacy' is merely an illusion of scale. The universe is a vast network of entangled information. We merely show you a glimpse of its true malleability."

Elara, who had been watching with fascination, suddenly pointed at the sphere. "Look! It's changing now! It's making a picture of a really fast spinning top!"

Alistair glanced back, his eyes widening. Indeed, the complex patterns within the water were now shifting, cohering into a mesmerizing, perfectly balanced image of a top, spinning with impossible, perpetual motion. It was not just an image; it conveyed the concept of ceaseless, self-sustaining rotation. It seemed to embody the very idea of infinite possibility within a closed system. Before Alistair could fully process this final, bewildering sight, a sudden, sharp jolt ran through the floor. The lights in the Atelier flickered, and a faint, distant hum of city traffic momentarily pierced the insulated silence. The air, which had been charged with an otherworldly energy, suddenly felt. . . ordinary.

The shopkeeper's serene expression remained, but they gestured subtly towards the now visible wooden door. "Ah. It seems our demonstration time for today has concluded. The Atelier, like certain quantum states, prefers not to be observed for too long in one location."

Alistair found himself propelled forward by an unseen force, or perhaps by his own desperate desire to escape. He stumbled out onto the street, blinking against the harsh reality of daylight. Elara was beside him, her hand still clutched in his. The street was bustling, just as it had been. He looked back.

The alleyway was empty. Where "The Atelier of Acausal Curiosities" had stood moments before, there was now only a grimy brick wall, stained with graffiti, and a dumpster overflowing with refuse. No door. No window. No sign.

"Where. . . where did it go?" Alistair stammered, looking wildly around.

Elara, however, was radiant. In her small hand, she held a simple, perfectly balanced spinning top, made of smooth, dark wood. It was utterly mundane, yet somehow, in her grasp, it seemed to hum with a faint, internal energy. She held it to her ear, a secret smile on her face.

"It whispers secrets, Uncle Alistair," she murmured, her eyes distant, filled with a knowledge far beyond her years. "Secrets of rearranged possibilities. It says the magic isn't in what you see, but in what you understand about what's hidden." Alistair looked at the spinning top, then back at the empty wall. His world of data, of algorithms, of predictable physics, felt utterly and irrevocably altered. He was left with the unsettling realization that his

meticulously ordered reality might be far more fluid, more deeply entangled, and infinitely more information rich than he had ever dared to imagine. The "magic" wasn't trickery, but a deeper, more unsettling truth about the nature of reality and the pervasive, manipulable influence of information, where entropy was not merely a law, but a landscape to be navigated, and perhaps, even rewritten. And he, Dr. Alistair Finch, the custodian of order, was now adrift in its boundless, beautiful, and terrifying chaos.

S4. Charts illustrating the data analysis of the "The Atelier of Acausal Curiosities," written by Gemini

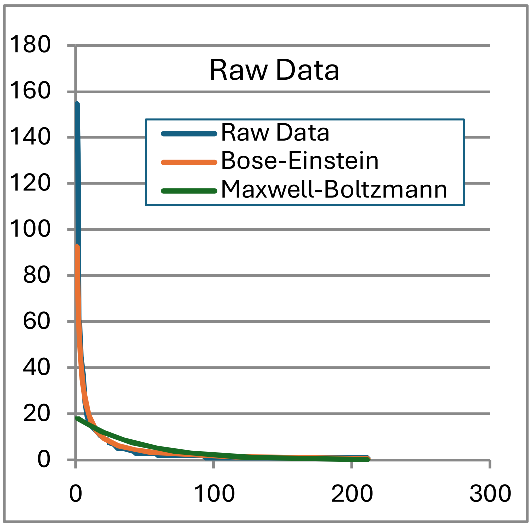

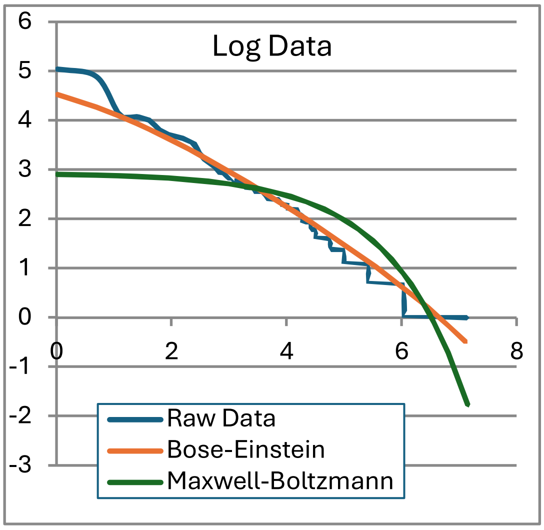

(a) **(b)**

Figure S3. (a) The 'numbers of appearances' $N(E_i)$ of words in "The Atelier of Acausal Curiosities," written by Gemini ranked from the lowest energy level, corresponding to the most frequently appearing word, to the highest energy level, corresponding to the least frequently appearing word,. The blue line represents the data, i.e. the numbers of appearances deduced from the story, the red line represents these numbers of appearances predicted by the BE distribution model, and same for the green line but for the MB distribution model. **(b)** The same data but using a log-log plot (both axes on a logarithmic scale). The red and blue line coincide almost completely in both graphs, whereas the green line does not coincide at all. This shows that the BE distribution is a good model for the numbers of appearances, while the MB distribution is not. We have $d = 0.75$.

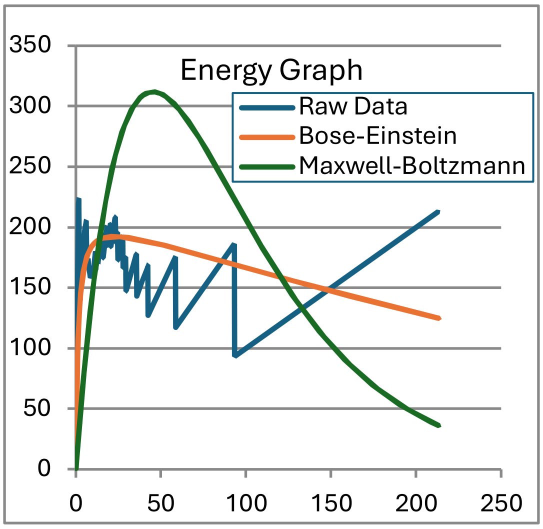

Figure S4. The energy $E(E_i)$ radiated per energy level of the "The Atelier of Acausal Curiosities," written by Gemini as a function of the energy levels E_i. The blue line represents the data, the red line represents the values predicted by the BE model, and same for the green line but for the MB model. We have $d = 0.75$.
